# High preoperative Glasgow prognostic score increases a risk of hospital mortality in elderly patients with perihilar cholangiocarcinoma

**DOI:** 10.1002/jhbp.12111

**Published:** 2025-02-13

**Authors:** Takashi Kokumai, Shuichi Aoki, Kei Nakagawa, Masahiro Iseki, Hideaki Sato, Takayuki Miura, Shimpei Maeda, Masaharu Ishida, Masamichi Mizuma, Michiaki Unno

**Affiliations:** ^1^ Department of Surgery Tohoku University Graduate School of Medicine Sendai Japan

**Keywords:** elderly patients, frailty, Glasgow prognostic score, perihilar cholangiocarcinoma, propensity score matching

## Abstract

**Background:**

Hemihepatectomy with extrahepatic bile duct resection is considered the only curative treatment for perihilar cholangiocarcinoma. The aim of the study was to clarify the survival benefits in this invasive surgical procedure for elderly patients.

**Methods:**

A total of 290 patients who underwent surgical resection for perihilar cholangiocarcinoma in our department from 2000 to 2020 were categorized into the E group (62 patients aged ≥75 years) and NE group (228 patients aged <74 years).

**Results:**

The E group exhibited decreased pathological lymph node metastasis (*p* = .001) and had a shorter operative time (*p* = .021) and fewer cases of combined vascular resection (*p* = .002). We found no significant differences in postoperative mortality; however, disease‐specific survival was significantly better in the E group (3‐year survival: 75.6 vs. 60.3%, *p* = .031). After propensity score matching, overall survival and disease‐specific survival did not differ between the two groups; nevertheless, the hospital mortality rate was significantly higher in the E group (11.5 vs. 1.6%, *p* = .020). In the E group, a high preoperative Glasgow prognostic score was the only factor associated with hospital mortality (odds ratio, 7.35; *p* = .026) and indicated worse prognosis.

**Conclusions:**

A high preoperative Glasgow prognostic score was associated with hospital mortality and poor prognosis in elderly patients with perihilar cholangiocarcinoma.

## Graphical abstract

Kokumai and colleagues demonstrated that surgical outcomes for elderly patients with perihilar cholangiocarcinoma are comparable to those for younger patients. However, propensity score matching revealed that a high preoperative Glasgow Prognostic Score is associated with increased hospital mortality and poor prognosis, highlighting the the need for thorough preoperative assessment.

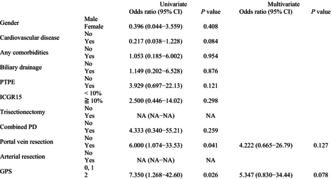

## 1 INTRODUCTION

Japan is one of the most aged societies worldwide. In Japan, the average human life expectancy was estimated to be 81.1 and 87.2 years for men and women in 2016, respectively. Furthermore, the proportion of elderly aged ≥65 years was 28.4% in 2019 and is estimated to reach 37.7% by 2050.^1^ A rapidly aging society markedly increases the demand for surgery among elderly patients with cancer.^2^ Elderly patients have age‐related issues that may increase their risk of hospital mortality or worsen their postoperative prognosis, such as poor performance status, diminished organ function, comorbidities, sarcopenia, and cognitive impairment.

Complete surgical resection through hemihepatectomy plus caudate lobectomy combined with extrahepatic bile duct resection provides a chance for cure in patients with resectable perihilar cholangiocarcinoma (PHCC).^3^, ^4^, ^5^ However, this surgical procedure requires the highest level of surgical technique and is highly invasive, with reported mortality rates ranging from 1.4% to 18%.^6^ Because of the high surgical difficulty and stress to patients with PHCC, the indication of surgical treatment for elderly patients with PHCC depends on the institution or the surgeon's experience. While several studies investigating the surgical outcomes of elderly patients with PHCC have verified the safety of hepatectomy,^7^, ^8^, ^9^ there exists a patient selection bias in determining the surgical approach. Although several scoring systems can be employed to predict postoperative mortality and morbidity,^10^, ^11^, ^12^ there is no evidence supporting the significant association of these systems with perioperative outcomes in elderly patients with PHCC.

Nutritional disorders with a loss of skeletal muscle and fat mass are considered an aging process and are caused by an elevated systemic inflammatory status. Recently, this nutritional status in patients with cancer has been recognized as “cancer‐induced weight loss” or “cachexia.”^13^ Cachexia negatively affects the therapeutic outcomes and leads to an adverse prognosis in patients with esophageal cancer, head and neck cancer, or pancreatic adenocarcinoma.^14^ Among the several scoring systems used to evaluate inflammation‐based nutritional status in patients with cancer, the Glasgow prognostic score (GPS), which is calculated based on C‐reactive protein (CRP) and serum albumin (Alb), is considered a useful tool that reflects systemic immune inflammatory status and predicts prognostic importance.^15^

The aim of the present study was to assess the importance of preoperative GPS for predicting postoperative death in elderly patients undergoing resection for PHCC. After a propensity score matching (PSM) analysis to eliminate the potential of patient's selection bias, we identify predictive factors for postoperative risk in elderly patients with PHCC.

## 2 METHODS

### 2.1 Study design

Data of 307 consecutive patients with PHCC who underwent major hepatectomy with extrahepatic bile duct resection at the Tohoku University Hospital (Sendai, Japan) from January 2000 to December 2020 were retrospectively collected. This study was performed in accordance with the principles embodied in the Declaration of Helsinki and was approved by our institutional review board (approval no.: 2016‐1‐061). The requirement for the acquisition of informed consent from patients was waived owing to the retrospective nature of this study.

### 2.2 Preoperative management

Preoperative management has been described in detail in our previous report.^3^ Briefly, computed tomography (CT), endoscopic ultrasonography, and endoscopic retrograde cholangiography were performed preoperatively. Patients with jaundice and/or dilated bile ducts in the remnant lobe routinely underwent endoscopic biliary drainage. Liver function was consistently evaluated using the indocyanine green (ICG) test. Preoperative percutaneous transhepatic portal embolization was performed when the volume of the future liver remnant was estimated to be less than 40% or when the future liver remnant plasma clearance rate of ICG (ICGK‐F) was below 0.05.^16^ The operative procedures were determined and planned via simulation using multidetector‐row CT.

### 2.3 Surgical treatment

All surgeries were performed after achieving a serum bilirubin level of <2 mg/dL. The surgical procedure performed for PHCC was hemihepatectomy or trisectionectomy plus caudate lobectomy combined with extrahepatic bile duct resection.^4^ The lymph nodes in the hepatoduodenal ligament, around the pancreatic head, and around the common hepatic artery were completely dissected; combined portal vein or hepatic artery resection was performed when the tumor invaded these vessels.^5^, ^17^ The resection margins of the bile duct were investigated for rapid intraoperative diagnosis; combined pancreatoduodenectomy was performed when needed to achieve curative resection.^17^ Pathological findings for the resected specimens were documented prospectively according to the Union for International Cancer Control's eighth TNM classification.

### 2.4 Postoperative follow‐up

Physical examination and blood tests, including those for tumor markers, were conducted every 3 months postoperatively. CT was performed at least twice per year for the first 5 years. Gemcitabine or S1 was administered as an adjuvant treatment, mainly for patients at a high risk for postoperative recurrence (e.g., lymph node metastasis).

### 2.5 Inflammation‐based nutritional assessment

The systemic inflammation‐based nutritional status before surgery was evaluated using well‐known scoring systems with nutritional and immunological factors—namely, the GPS, controlling nutritional status (CONUT) score, neutrophil‐to‐lymphocyte ratio (NLR), platelet‐to‐lymphocyte ratio (PLR), and Alb‐to‐globulin ratio (AGR).^18^, ^19^, ^20^, ^21^, ^22^, ^23^ Blood samples were obtained a few days before surgery. No clinical evidence of infection or other inflammatory conditions was observed at the time of blood sampling. The GPS was defined as follows: (i) patients with normal Alb level ≥3.5 g/dL and CRP level ≤1.0 mg/dL were scored as 0, (ii) those with low Alb level <3.5 g/dL or high CRP level >1.0 mg/dL were scored as 1, and (iii) those with low Alb level <3.5 g/dL and high CRP level >1.0 mg/dL were scored as 2, with a GPS of 2 indicating malnutrition. The CONUT score was defined as follows: (i) patients with Alb levels ≥3.5 g/dL, 3.0–3.49 g/dL, 2.5–2.99 g/dL, and <2.5 g/dL were scored as 0, 2, 4, and 6, respectively; (ii) patients with total cholesterol levels ≥180 mg/dL, 140–179 mg/dL, 100–139 mg/dL, and <100 mg/dL were scored as 0, 1, 2, and 3, respectively; and (iii) patients with lymphocyte counts ≥1600/μL, 1200–1599/μL, 800–1199/μL, and < 800/μL were scored as 0, 1, 2, and 3, respectively. The CONUT score was calculated as the summation of all scores, with a score of ≥5 indicating malnutrition. The NLR, PLR, and AGR were calculated by dividing the neutrophil count by the lymphocyte count, the platelet count by the lymphocyte count, and the Alb level by the globulin level.

### 2.6 Statistical analysis

Categorical and continuous variables were examined using the chi‐square test and Mann–Whitney U test, respectively. A PSM analysis was conducted to adjust for confounding clinicopathological and intraoperative factors. An adjustment of variables was made for patients' background and surgical factors showing significant between‐group differences in all cases, including operative procedures, vascular resection, and pathological lymph node metastases. For the PSM analysis, one‐to‐one matching was performed between the groups using the nearest‐neighbor matching method with a caliper width of 0.2. Survival curves were constructed using the Kaplan Meier method and compared using the log‐rank test. Univariate and multivariate analyses were performed using a Cox proportional hazards regression model in a stepwise fashion. Odds ratios (OR) and 95% confidence intervals (CI) were calculated for each category. All statistical analyses were performed using JMP Pro version 16.0.0 (SAS Institute Inc., Cary, NC) and GraphPad Prism version 10.0.2 (GraphPad Software, San Diego, CA), with statistical significance set at *p* < .05.

## 3 RESULTS

### 3.1 Patient selection and clinicopathological factors

**FIGURE 1:**
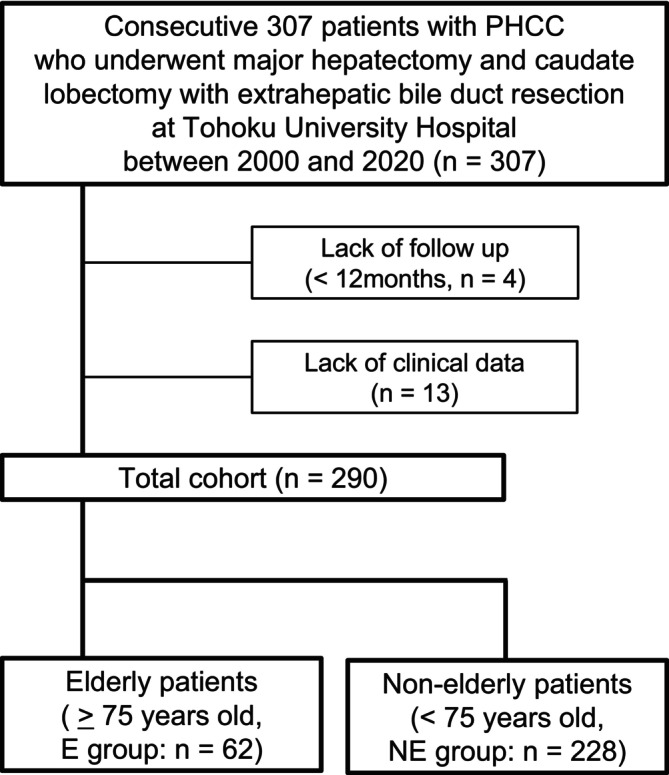
Flow chart of the patient selection. A total of 307 patients with perihilar cholangiocarcinoma underwent major hepatectomy with extrahepatic bile duct resection. Out of these 307 patients, four who were lost to postoperative follow‐up within 1 year and 13 without clinical data were excluded from this study. Finally, a total of 290 patients were categorized into two groups: The elderly (E) group (62 patients aged >75 years) and the non‐elderly (NE) group (228 patients aged <74 years).

Among 307 consecutive patients with PHCC who underwent major hepatectomy with extrahepatic bile duct resection at the Tohoku University Hospital from 2000 to 2020, four patients who were lost to postoperative follow‐up within 1 year and 13 patients without clinical data were excluded from the study. Ultimately, 290 patients were included in this study and categorized into two groups: the elderly (E) group (62 patients aged ≥75 years) and the non‐elderly (NE) group (228 patients aged <74 years) (Figure 1). Table 1 summarizes the clinicopathological characteristics of the cohort.

**TABLE 1:** Descriptive statistics for the total cohort.

	Total cohort
Number	290
Gender, *n* (%)	
Male	211 (72.8%)
Female	79 (27.2%)
Age (year)	
Median (range)	69 (28–85)
Preoperative biliary drainage, *n* (%)	
Yes	236 (81.4%)
No	54 (18.6%)
PTPE, *n* (%)	
Yes	142 (49.0%)
No	148 (51.0%)
ICG R15 (%)	
Median (range)	8.60 (0.80–61.8)
Type of hepatectomy, *n* (%)	
Right hepatectomy	135 (46.5%)
Left hepatectomy	99 (34.1%)
Right‐sided trisegmentectomy	7 (2.4%)
Left‐sided trisegmentectomy	17 (5.9%)
HPD (right)	17 (5.9%)
HPD (left)	15 (5.2%)
Combined portal vein resection, *n* (%)	
Yes	86 (29.7%)
No	204 (70.3%)
Combined arterial resection, *n* (%)	
Yes	10 (3.4%)
No	280 (96.6%)
Blood loss (mL)	
Median (range)	1664 (355–56 331)
Operation time (minutes)	
Median (range)	640 (139–1165)
Blood transfusion, *n* (%)	
Yes	142 (49.0%)
No	148 (51.0%)
Histopathological grade, *n* (%)	
Well differentiated adenocarcinoma	75 (25.9%)
Moderately differentiated adenocarcinoma	195 (67.2%)
Poorly differentiated adenocarcinoma	20 (6.9%)
T classification (UICC), *n* (%)	
0/1	16 (5.5%)
2	146 (50.4%)
3	38 (13.1%)
4	90 (31.0%)
Pathological portal vein invasion, *n* (%)	
Yes	76 (26.2%)
No	213 (73.8%)
Pathological arterial invasion, *n* (%)	
Yes	30 (10.3%)
No	260 (89.7%)
Bismuth, *n* (%)	
I	11 (3.8%)
II	57 (19.7%)
III	132 (45.5%)
IV	90 (31.0%)
N classification (UICC), *n* (%)	
0	161 (55.5%)
1	129 (44.5%)
Stage classification (UICC), *n* (%)	
0/I	16 (5.5%)
II	78 (26.9%)
III	82 (28.3%)
IV	114 (39.3%)
Pathological lymphatic invasion, *n* (%)	
No	63 (21.7%)
Yes	227 (78.3%)
Pathological vessel invasion, *n* (%)	
No	40 (13.8%)
Yes	250 (86.2%)
Pathological perineural invasion, *n* (%)	
No	41 (14.1%)
Yes	249 (85.9%)
Residual tumor, *n* (%)	
R0	203 (70.0%)
R1	83 (28.6%)
R2	4 (1.4%)
SSI, *n* (%)	
Superficial/deep	85 (29.3%)
Organ/space	129 (44.5%)
Abdominal abscess, *n* (%)	
No	181 (62.4%)
Yes	109 (37.6%)
Bile leakage, *n* (%)	
No	203 (70.0%)
Yes	87 (30.0%)
Pancreatic fistula (ISGPF), *n* (%)	
No/biochemical leak	262 (90.4%)
Grade B	23 (7.9%)
Grade C	5 (1.7%)
Clavien‐Dindo classification, *n* (%)	
0	36 (12.4%)
I	33 (11.4%)
II	57 (19.7%)
III	113 (39.0%)
IV	25 (8.6%)
V	26 (8.9%)
Hospital stays (days)	
Median (range)	35 (3–220)
Hospital death (≦90 days), *n* (%)	
No	268 (92.4%)
Yes	22 (7.6%)

Abbreviations: HPD, hepatopancreatoduodenectomy; ICGR15, indocyanine green retention at 15 min; ISGPF, International study group of postoperative pancreatic fistula; PTPE, percutaneous transhepatic portal vein embolization; SSI, surgical site infection; UICC, Union for International Cancer Control.

### 3.2 Association between clinicopathological factors before and after PSM

Clinicopathological factors were compared between the E and NE groups (Table 2). With respect to the intraoperative factors, patients in the E group tended to undergo left hepatectomy (50.0% vs. 36.4%, *p* = .060) rather than right hepatectomy or trisectionectomy. Combined pancreatoduodenectomy was performed less frequently in the E group (4.8% vs. 12.7%, *p* = .057). The E group patients were associated with shorter operative times (median time: 585 vs 651 min, *p* = .021) and were less likely to undergo combined portal vein resection compared to NE group patients (85.5% vs. 66.2%, *p* = .002). Pathologically, the E group patients were less likely to have lymph node metastasis (49.6% vs. 25.8%, *p* = .001) and advanced‐stage disease (41.9% vs. 29.8%, *p* = .075). PSM was conducted to mitigate these between‐group differences by matching 61 out of 62 patients in the E group (E‐PSM group) with 61 out of 228 patients in the NE group (NE‐PSM group). After PSM, no significant differences in descriptive variables and clinicopathological factors were found between the two groups (Table 2).

**TABLE 2:** Association between clinicopathological factors and patient age before and after propensity score matching (PSM).

	Before PSM		*p*‐value	After PSM		*p*‐value
	Elderly patients (E group)	Non‐elderly patients (NE group)		Elderly patients (E‐PSM group)	Non‐elderly patients (NE‐PSM group)	
Number	62	228		61	61	
Gender, (*n*)						
Male:Female	45: 17	166: 62	.972	44: 17	44: 17	1.000
Age (year)						
Median (range)	77 (75–85)	67 (28–74)	<.001	77 (75–85)	67 (47–74)	<.001
Biliary drainage, *n* (%)						
Yes	43 (69.4%)	193 (84.7%)	.009	42 (68.9%)	45 (73.8%)	.548
No	19 (30.6%)	35 (15.3%)		19 (31.1%)	16 (26.2%)	
PTPE, *n* (%)						
Yes	26 (41.9%)	116 (50.9%)	.211	26 (42.6%)	25 (41.0%)	.854
No	36 (58.1%)	112 (49.1%)		35 (57.4%)	36 (59.0%)	
ICGR15 (%)						
Median (range)	10.5 (3.50–61.8)	8.3 (0.80–37.8)	.109	10.6 (3.50–61.8)	8.3 (2.30–37.8)	.163
Type of hepatectomy, *n* (%)						
Right hepatectomy	29 (46.8%)	123 (54.0%)	.060	29 (47.5%)	25 (41.0%)	.303
Left hepatectomy	31 (50.0%)	83 (36.4%)		30 (49.2%)	30 (49.2%)	
Trisectionectomy	2 (3.2%)	22 (9.6%)		2 (3.3%)	6 (9.8%)	
Combined PD, *n* (%)						
Yes	3 (4.8%)	29 (12.7%)	.057	3 (4.9%)	7 (11.5%)	.181
No	59 (95.2%)	199 (87.3%)		58 (95.1%)	54 (88.5%)	
Portal vein resection, *n* (%)						
Yes	9 (14.5%)	77 (33.8%)	.002	9 (14.8%)	9 (14.8%)	1.000
No	53 (85.5%)	151 (66.2%)		52 (85.2%)	52 (85.2%)	
Arterial resection, *n* (%)						
Yes	1 (1.6%)	9 (4.0%)	.332	1 (1.6%)	1 (1.6%)	1.000
No	61 (98.4%)	219 (96.0%)		60 (98.4%)	60 (98.4%)	
Blood loss (mL)						
Median (range)	1615 (415–7080)	1693 (355–56 331)	.095	1620 (415–7080)	1627 (715–8130)	.116
Operation time (minutes)						
Median (range)	585 (374–982)	651 (139–1165)	.021	584 (374–982)	647 (139–1165)	.180
Blood transfusion, *n* (%)						
Yes	32 (51.6%)	110 (48.3%)	.638	32 (52.5%)	29 (47.5%)	.587
No	30 (48.4%)	118 (51.7%)		29 (47.5%)	32 (52.5%)	
Well differentiated, *n* (%)						
Yes	17 (27.4%)	58 (25.4%)	.753	17 (27.9%)	17 (27.9%)	1.000
No	45 (72.6%)	170 (74.6%)		44 (72.1%)	44 (72.1%)	
T classification (UICC), *n* (%)						
0, 1, 2	37 (59.7%)	123 (54.0%)	.420	37 (60.7%)	41 (67.2%)	.451
3, 4	25 (40.3%)	105 (46.0%)		24 (39.3%)	20 (32.8%)	
Portal vein invasion, *n* (%)						
Yes	14 (22.6%)	62 (27.3%)	.448	14 (23.0%)	10 (16.4%)	.361
No	48 (77.4%)	165 (72.7%)		47 (77.0%)	51 (83.6%)	
Arterial invasion, *n* (%)						
Yes	7 (11.3%)	23 (10.1%)	.785	7 (11.5%)	3 (4.9%)	.181
No	55 (88.7%)	205 (89.9%)		54 (88.5%)	58 (95.1%)	
Bismuth, *n* (%)						
I–II	16 (25.8%)	52 (22.8%)	.881	16 (26.2%)	16 (26.2%)	.978
III	27 (43.6%)	105 (46.0%)		27 (44.3%)	28 (45.9%)	
IV	19 (30.6%)	71 (31.2%)		18 (29.5%)	17 (27.9%)	
*N* classification (UICC), *n* (%)						
0	46 (74.2%)	115 (50.4%)	.001	45 (73.8%)	45 (73.8%)	1.000
1	16 (25.8%)	113 (49.6%)		16 (26.2%)	16 (26.2%)	
Stage classification (UICC), *n* (%)						
0–II	26 (41.9%)	68 (29.8%)	.075	26 (42.6%)	29 (47.5%)	.585
III–IV	36 (58.1%)	160 (70.2%)		35 (57.4%)	32 (52.5%)	
Lymphatic vessel invasion, *n* (%)						
No	20 (32.3%)	43 (18.9%)	.028	20 (32.8%)	16 (26.2%)	.427
Yes	42 (67.7%)	185 (81.1%)		41 (67.2%)	45 (73.8%)	
Blood vessel invasion, *n* (%)						
No	8 (12.9%)	32 (14.0%)	.818	8 (13.1%)	12 (19.7%)	.327
Yes	54 (87.1%)	196 (86.0%)		53 (86.9%)	49 (80.3%)	
Perineural invasion, *n* (%)						
No	12 (19.4%)	29 (12.7%)	.198	12 (19.7%)	13 (21.3%)	.823
Yes	50 (80.6%)	199 (87.3%)		49 (80.3%)	48 (78.7%)	
Residual tumor, *n* (%)						
R0	43 (69.4%)	160 (70.2%)	.981	43 (70.5%)	43 (70.5%)	1.000
R1	18 (29.0%)	65 (28.5%)		18 (29.5%)	18 (29.5%)	
R2	1 (1.6%)	3 (1.3%)		0 (0.0%)	0 (0.0%)	

Abbreviations: PSM, propensity score matching; PTPE, percutaneous transhepatic portal vein embolization; ICGR15, indocyanine green retention at 15 min; PD, pancreatoduodenectomy; UICC, Union for International Cancer Control.

### 3.3 Short‐term outcomes analysis stratified by patient age before and after PSM

Postoperative complications before and after PSM were compared between the two groups (Table 3). The two groups did not significantly differ in terms of surgical site infection, abdominal abscess, pancreatic fistula, postoperative complications with Clavien‐Dindo grade III or higher, and length of hospital stay before and after PSM. Interestingly, the rates of bile leakage tended to be higher in the E‐PSM group (36.1% vs. 21.3%, *p* = .070). Hospital death within 90 days after surgery was significantly higher in the E‐PSM group (11.5% vs. 1.6%, *p* = .020); in contrast, there was no significant difference between the E and NE groups before PSM.

**TABLE 3:** Association between postoperative complications and patient age determined in before and after propensity score matching.

	Before PSM		*p*‐value	After PSM		*P*‐value
	Elderly patients (E group)	Non‐elderly patients (NE group)		Elderly patients (E‐PSM group)	Non‐elderly patients (NE‐PSM group)	
Number	62	228		61	61	
SSI, *n* (%)						
Superficial/deep	20 (32.3%)	65 (28.5%)	.934	20 (32.8%)	18 (29.5%)	.276
Organ/space	31 (50.0%)	98 (43.0%)		31 (50.8%)	23 (37.7%)	
Abdominal abscess, *n* (%)						
No	37 (59.7%)	84 (36.8%)	.617	36 (59.0%)	43 (70.5%)	.184
Yes	25 (40.3%)	144 (63.2%)		25 (41.0%)	18 (29.5%)	
Bile leakage, *n* (%)						
No	40 (64.5%)	163 (71.5%)	.293	39 (63.9%)	48 (78.7%)	.070
Yes	22 (35.5%)	65 (28.5%)		22 (36.1%)	13 (21.3%)	
Pancreatic fistula (ISGPF), *n* (%)						
No/biochemical leak	57 (91.9%)	205 (89.9%)	.626	56 (91.8%)	57 (93.4%)	.729
Grade B/C	5 (8.1%)	23 (10.1%)		5 (8.2%)	4 (6.6%)	
Clavien‐Dindo classification, *n* (%)						
0–II	26 (41.9%)	100 (43.9%)	.786	25 (41.0%)	32 (52.5%)	.204
III–V	36 (58.1%)	128 (56.1%)		36 (59.0%)	29 (47.5%)	
Hospital stays (days)						
Median (range)	34 (3–150)	35 (9–220)	.615	35 (3–150)	39 (16–169)	.412
Hospital death (≦90 days), *n* (%)						
No	55 (88.7%)	213 (93.4%)	.235	54 (88.5%)	60 (98.4%)	.020
Yes	7 (11.3%)	15 (6.6%)		7 (11.5%)	1 (1.6%)	

Abbreviations: ISGPF, International study group of postoperative pancreatic fistula; PSM, propensity score matching; SSI, surgical site infection.

### 3.4 Long‐term outcomes analysis stratified by patient age before and after PSM

**FIGURE 2:**
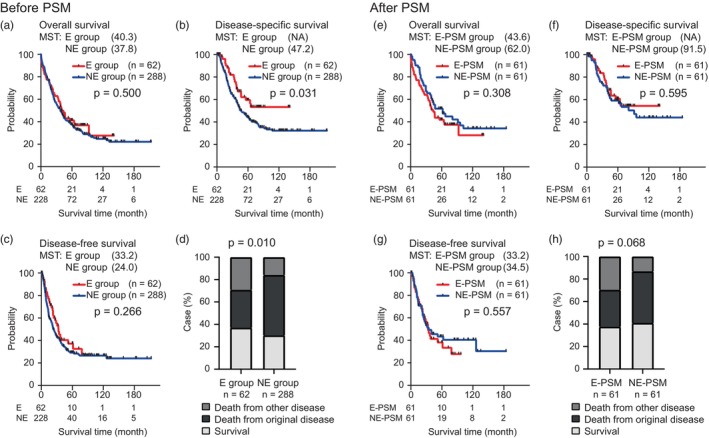
(a–d) Survival analysis stratified by patient age before propensity score matching (PSM): E and NE groups. (a) Overall survival (OS), (b) disease‐specific survival (DSS), and (c) disease‐free survival (DFS). (d) Comparison of outcomes classified as survival, death from original disease, and death from other disease. (e–h) Survival analysis stratified by patient age after PSM: E‐PSM and NE‐PSM groups. (e) OS, (f) DSS, and (g) DFS. (h) Comparison of outcomes classified as survival, death from original disease, and death from other disease. MST, median survival time; E, elderly; NE, non‐elderly; NA, not applicable.

Among 290 patients who underwent major hepatectomy with extrahepatic bile duct resection for PHCC, the median overall survival (OS), disease‐specific survival (DSS), and disease‐free survival (DFS) were 39.5, 53.6, and 26.4 months, respectively. Survival analysis stratified by patient age indicated no significant differences in OS and DFS (Figure 2a,c). However, the E group showed significantly better DSS than the NE group (median DSS: not applicable vs. 47.2 months, 3‐year survival: 75.6 vs. 60.3%, *p* = .031; Figure 2b).

Regarding the cause of death in the two groups, more patients in the E group died of other diseases, not the original diseases, than those in the NE group (29.0% vs. 15.8%, *p* = .010; Figure 2d). Among 122 patients after PSM, the median OS, DSS, and DFS were 46.7, 91.5, and 34.4 months, respectively. Survival analysis stratified by patient age indicated no significant differences in OS, DSS, and DFS (Figure 2e–g). The causes of death in the two groups were similar to those before PSM (survival/death from the original diseases/death from other diseases: 37.7%/32.8%/29.5% vs. 41.0%/45.9%/13.1%, respectively, *p* = .068; Figure 2h).

### 3.5 Association between preoperative nutrition and hospital death

Preoperative nutrition associated with 90‐day postoperative mortality was assessed in the E‐PSM group (Table S1). A preoperative GPS of 2 was significantly associated with death within 90 days after surgery (42.9% vs. 9.3%, *p* = .033). No association was observed between preoperative malnutrition and 90‐day postoperative mortality according to the Alb, CRP, CONUT score, NLR, PLR, and AGR. In the NE group, none of the clinical factors, including nutritional status, were associated with 90‐day postoperative mortality (Table S2).

### 3.6 Univariate and multivariate analyses using the Cox proportional hazards model

Univariate analysis was conducted to clarify the preoperative factors influencing 90‐day postoperative mortality in the E‐PSM group (Table 4). A preoperative GPS of 2 (odds ratio [OR] 7.350; 95% CI: 1.268–42.60; *p* = .026) and portal vein resection (OR 6.000; 95% CI: 1.074–33.53; *p* = .041) were significantly associated with 90‐day postoperative mortality. Multivariate analysis showed a preoperative GPS of 2 had a limited effect on an independent predictor of 90‐day postoperative mortality (OR 5.374; 95% CI: 0.830–34.44; *p* = .078).

**TABLE 4:** Univariate and multivariate analysis for preoperative factors associated with hospital death in elderly patients.

		Univariate		Multivariate	
		Odds ratio	*p*‐value	Odds ratio	*p*‐value
		(95% CI)		(95% CI)	
Gender	Male				
	Female	0.396 (0.044–3.559)	.408		
Cardiovascular disease	No				
	Yes	0.217 (0.038–1.228)	.084		
Any comorbidities	No				
	Yes	1.053 (0.185–6.002)	.954		
Biliary drainage	No				
	Yes	1.149 (0.202–6.528)	.876		
PTPE	No				
	Yes	3.929 (0.697–22.13)	.121		
ICGR15	<10%				
	≧10%	2.500 (0.446–14.02)	.298		
Trisectionectomy	No				
	Yes	NA (NA–NA)	NA		
Combined PD	No				
	Yes	4.333 (0.340–55.21)	.259		
Portal vein resection	No				
	Yes	6.000 (1.074–33.53)	.041	4.222 (0.665–26.79)	.127
Arterial resection	No				
	Yes	NA (NA–NA)	NA		
GPS	0, 1				
	2	7.350 (1.268–42.60)	.026	5.347 (0.830–34.44)	.078

Abbreviations: CI, confidence interval; GPS, Glasgow prognostic score; ICGR15, indocyanine green retention at 15 min; NA, not applicable; PD, pancreatoduodenectomy; PTPE, percutaneous transhepatic portal vein embolization.

**FIGURE 3:**
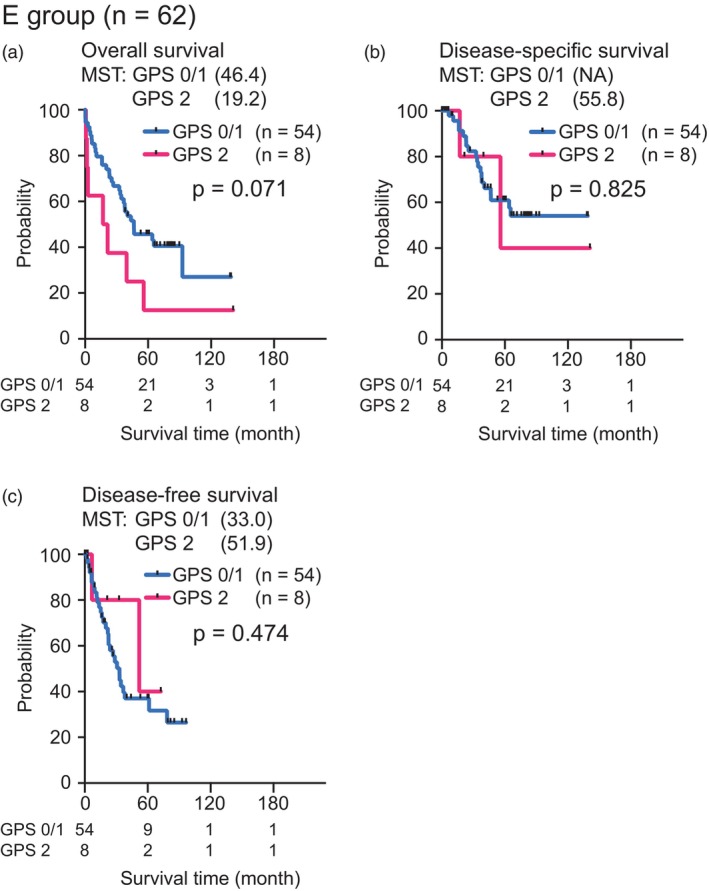
Survival analysis stratified by Glasgow prognostic score (GPS) in elderly patients before propensity score matching. (a) Overall survival, (b) disease‐specific survival, and (c) disease‐free survival. MST, median survival time; NA, not applicable.

Survival analysis stratified by the GPS in elderly patients revealed that the E group with a GPS of 0/1 tended to have better OS than the E group with a GPS of 2 (median OS: 46.4 vs. 19.2 months, *p* = .071) (Figure 3a). There were no significant differences in DSS or DFS between the two groups (Figure 3b,c).

## 4 DISCUSSION

Among several studies investigating the surgical outcomes of elderly patients with PHCC,^7^, ^8^, ^9^ two reports were from the Nagoya University in Japan, which is one of the world's leading high‐volume centers for PHCC surgery. With a large number of cases, these reports found no significant differences in short‐ and long‐term prognoses between patients with PHCC aged ≥80 years and younger patients. However, there was a selection bias in the patients introduced to the institution and in the candidates for surgical treatments. Thus, most elderly patients who underwent surgery in these reports were expected unintentionally beyond the strict indication for invasive surgical treatment, reflecting the predominance of less advanced tumors in elderly patients. Similarly, the current study also showed that elderly patients who underwent surgery had less advanced tumors and did not require vascular resection. This suggests that elderly patients with resectable PHCC may have been deemed contraindicated for definitive surgery due to age‐related comorbidities or a decline in general health following preoperative management, such as biliary drainage; therefore, perioperative mortality was guaranteed, and DSS was significantly better in elderly patients than in younger patients. Conversely, more elderly patients died of other diseases, resulting in an OS comparable to that of younger patients. Notably, we performed a PSM analysis to eliminate the influence of selection bias and observed a higher risk of perioperative mortality in elderly patients. Furthermore, we showed that a poor preoperative GPS was a useful surrogate marker for assessing the risk of perioperative mortality and that elderly patients who could tolerate PHCC surgery might benefit from survival, which corresponds to that of younger patients.

Among the various assessments of systemic inflammation‐based nutritional status, such as the CONUT score, NLR, PLR, AGR, and GPS, the GPS, which is calculated based on CRP and Alb, was the most appropriate nutritional assessment that was significantly associated with postoperative outcomes. While both CRP and Alb are recognized as biomarkers for nutritional status and inflammation, neither parameter alone showed significance in our study. However, GPS effectively highlighted the implications for postoperative mortality by combining these markers. The GPS provides a more comprehensive overview of a patient's nutritional and inflammatory status compared to any individual assessment. Furthermore, this scoring system can differentiate between varying stages of cachexia and is a significant predictor of therapeutic responsiveness.^24^

Alb is a protein produced by the liver that is used in the various assessments of nutritional status. CRP is recognized as one of the acute‐phase proteins produced by interleukin (IL)‐6, one of the proinflammatory cytokines. Proinflammatory cytokines are activated and released through the tumor‐mediated pathway in the regional tumor area and can lead to systemic metabolic changes, resulting in a hypercatabolic state and muscle degradation, ultimately causing malnutrition.^25^ In the patients with PHCC, effective preoperative management aimed at improving liver function and nutritional status, including percutaneous transhepatic portal embolization and treatment of cholangitis, is crucial for ensuring postoperative safety. Poor preoperative management likely contribute to elevated systemic inflammation, malnutrition and reduced liver function, and increased rate of the postoperative death, emphasizing the need for comprehensive preoperative interventions, particularly in elderly patients. Therefore, the preoperative assessment using GPS was directly related to postoperative short‐term outcome, as well as specific surgical procedures, including combined portal vein resection or various types of hepatectomy.

While the nutrition status and surgical procedure were significantly associated with hospital death in elderly patients, this association was not observed in non‐elderly patients. Elderly patients commonly have multiple comorbidities and frailer health status, making them more vulnerable to surgical stress and increasing their risk of postoperative complications. In fact, this current study showed the relatively higher incidence of biliary leakage and hospital mortality in elderly patients. The GPS potentially reflect the frailty of elderly patients and serve as a predictor of their outcomes. In contrast, non‐elderly patients generally have fewer age‐related comorbidities and tend to present with better performance status and overall health, reducing their risk of perioperative mortality. The 15 cases of hospital death in the NE group may have resulted from other factors such as acute physiological responses to invasive surgery or infections acquired postoperatively, underlying health variability that are less influenced by nutritional and systemic inflammatory status. Further investigation into the specific causes of mortality in non‐elderly patients would provide a deeper understanding of these differences and inform management strategies for this demographic.

It is important to preoperatively assess the frailty of patients with PHCC to estimate postoperative short‐ and long‐term outcomes because their poor performance status or impaired organ function can significantly hinder tolerance to the invasiveness of surgical treatment. “Fit patients” can obtain the therapeutic benefit from standard treatment, whereas “frail patients” have a poor prognosis.^26^ Although various parameters related with performance status, nutrition, and comorbidities are used as geriatric assessments,^27^ the current study proposed that the GPS may be a useful tool for correctly identifying “frail patients” with PHCC.

This study had several limitations. First, the results were based on retrospectively collected data from a single‐center study, including the confounding factors and a selection bias. The cohort size was small, making it difficult to identify the factors associated with 90‐day postoperative mortality using multivariate analysis. Second, we did not strictly manage nutritional support during the preoperative treatment; therefore, this bias potentially influenced the preoperative inflammation‐based nutritional status and survival differences. Third, indications were not strictly controlled because of the retrospective nature of the study. Further prospective studies are required to address this issue.

In conclusion, the short‐ and long‐term outcomes of elderly patients with PHCC do not differ from those of younger patients in our department; however, a poor preoperative GPS implies a risk of hospital death and poor prognosis. To stratify the frailty in elderly patients, a high GPS should be carefully determined for the indications for surgery in elderly PHCC patients.

## CONFLICT OF INTEREST STATEMENT

The authors declare no conflict of interest for this article.

## Supporting information


Data S1.
