# Supplementary material for: High preoperative Glasgow prognostic score increases a risk of hospital mortality in elderly patients with perihilar cholangiocarcinoma
Source: J Hepatobiliary Pancreat Sci. 2025 Feb 13;32(4):298–310. doi: 10.1002/jhbp.12111 (PMC12038378; doi:10.1002/jhbp.12111)
Supplement: Supplementary file 1 — Data S1. [file JHBP-32-298-s001.docx]

| **Supplementary table 1**. Association between operative factors and hospital death in elderly patients | | | | |
| --- | --- | --- | --- | --- |
|  | | Elderly patients (E-PSM group) | | *p* value |
|  | | Hospital death (≦ 90 days) | Other patients |  |
| Number | | 7 | 54 |  |
| Gender, n (%) | |  | | |
|  | Male | 6 (85.7%) | 38 (70.4%) | 0.366 |
|  | Female | 1 (14.3%) | 16 (29.6%) |  |
| Cardiovascular disease, n (%) | |  | | |
|  | Yes | 2 (28.6%) | 35 (64.8%) | 0.067 |
|  | No | 5 (71.4%) | 23 (35.2%) |  |
| Respiratory disease, n (%) | |  | | |
|  | Yes | 1 (14.3%) | 1 (1.9%) | 0.168 |
|  | No | 6 (85.7%) | 53 (98.1%) |  |
| Diabetes mellitus, n (%) | |  | | |
|  | Yes | 1 (14.3%) | 12 (22.2%) | 0.615 |
|  | No | 6 (85.7%) | 42 (77.8%) |  |
| Anticoagulant drug, n (%) | |  | | |
|  | Yes | 0 (0.0%) | 10 (18.5%) | 0.102 |
|  | No | 7 (100.0%) | 44 (81.5%) |  |
| No comorbidity, n (%) | |  | | |
|  | Yes | 2 (28.6%) | 16 (29.6%) | 0.954 |
|  | No | 5 (71.4%) | 38 (70.4%) |  |
| Biliary drainage, n (%) | |  | | |
|  | Yes | 5 (71.4%) | 37 (68.5%) | 0.875 |
|  | No | 2 (28.6%) | 17 (31.5%) |  |
| PTPE, n (%) | |  | | |
|  | Yes | 5 (71.4%) | 21 (38.9%) | 0.101 |
|  | No | 2 (28.6%) | 33 (61.1%) |  |
| ICGR15 (%) | |  | | |
|  | Median (range) | 12.00 (9.50−39.0) | 10.5 (3.50−61.8) | 0.335 |
| Type of hepatectomy, n (%) | |  | | |
|  | Right-hepatectomy | 6 (85.7%) | 23 (42.6%) | 0.077 |
|  | Left-hepatectomy | 1 (14.3%) | 29 (53.7%) |  |
|  | Trisectionectomy | 0 (0.0%) | 2 (3.7%) |  |
| Combined PD, n (%) | |  | | |
|  | Yes | 1 (14.3%) | 2 (3.7%) | 0.300 |
|  | No | 6 (85.7%) | 52 (96.3%) |  |
| Portal vein resection, n (%) | |  | | |
|  | Yes | 3 (42.9%) | 6 (11.1%) | 0.051 |
|  | No | 4 (57.1%) | 48 (88.9%) |  |
| Arterial resection, n (%) | |  | | |
|  | Yes | 0 (0.0%) | 1 (1.9%) | 0.620 |
|  | No | 7 (100.0%) | 53 (98.1%) |  |
| Alb, n (%) | |  | | |
|  | ≧3.5 | 2 (28.6%) | 31 (57.4%) | 0.147 |
|  | <3.5 | 5 (71.4%) | 23 (42.6%) |  |
| CRP, n (%) | |  | | |
|  | ≦1.0 | 4 (57.1%) | 46 (85.2%) | 0.100 |
|  | >1.0 | 3 (42.9%) | 8 (14.8%) |  |
| GPS, n (%) | |  | | |
|  | 0, 1 | 4 (57.1%) | 49 (90.7%) | 0.033 |
|  | 2 | 3 (42.9%) | 5 (9.3%) |  |
| CONUT score, n (%) | |  | | |
|  | 0-4 | 5 (71.4%) | 45 (83.3%) | 0.465 |
|  | 5-12 | 2 (28.6%) | 9 (16.7%) |  |
| NLR | |  | | |
|  | Median (range) | 2.069 (0.891−6.92) | 2.025 (0.800−8.000) | 0.885 |
| PLR | |  | | |
|  | Median (range) | 132.4 (82.38−450.4) | 148.2 (43.20−347.4) | 0.832 |
| AGR | |  | | |
|  | Median (range) | 0.810 (0.491−1.219) | 1.075 (0.660−1.483) | 0.104 |
| Abbreviations are PTPE, percutaneous transhepatic portal vein embolization; ICGR15, indocyanine green retention at 15 min; PD, pancreatoduodenectomy; Alb, serum albumin; CRP, C-reactive protein; GPS, Glasgow prognostic score; CONUT, controlling nutritional status; NLR, neutrophil-lymphocyte ratio; PLR, platelet-lymphocyte ratio; AGR, albumin-globulin ratio. | | | | |

| **Supplementary table 2**. Association between operative factors and hospital death in non-elderly patients | | | | |
| --- | --- | --- | --- | --- |
|  | | Non-elderly patients (NE group) | | *p* value |
|  | | 90-day mortality | Other patients |  |
| Number | | 15 | 213 |  |
| Gender, n (%) | |  | | |
|  | Male | 11 (73.3%) | 155 (72.8%) | 0.962 |
|  | Female | 4 (26.7%) | 58 (27.2%) |  |
| Biliary drainage, n (%) | |  | | |
|  | Yes | 14 (93.3%) | 179 (84.0%) | 0.288 |
|  | No | 1 (6.7%) | 34 (16.0%) |  |
| PTPE, n (%) | |  | | |
|  | Yes | 8 (53.3%) | 108 (50.7%) | 0.844 |
|  | No | 7 (46.7%) | 105 (49.3%) |  |
| ICGR15 (%) | |  | | |
|  | Median (range) | 9.00 (2.00−20.6) | 8.30 (0.80−37.8) | 0.980 |
| Type of hepatectomy, n (%) | |  | | |
|  | Right-hepatectomy | 9 (60.0%) | 114 (53.5%) | 0.235 |
|  | Left-hepatectomy | 3 (20.0%) | 80 (37.6%) |  |
|  | Trisectionectomy | 3 (20.0%) | 19 (8.9%) |  |
| Combined PD, n (%) | |  | | |
|  | Yes | 2 (13.3%) | 27 (12.7%) | 0.942 |
|  | No | 13 (86.7%) | 186 (87.3%) |  |
| Portal vein resection, n (%) | |  | | |
|  | Yes | 7 (46.7%) | 70 (32.9%) | 0.285 |
|  | No | 8 (53.3%) | 143 (67.1%) |  |
| Arterial resection, n (%) | |  | | |
|  | Yes | 0 (0.0%) | 9 (4.2%) | 0.263 |
|  | No | 15 (100.0%) | 204 (95.8%) |  |
| Alb, n (%) | |  | | |
|  | ≧3.5 | 6 (40.0%) | 124 (58.2%) | 0.171 |
|  | <3.5 | 9 (60.0%) | 89 (41.8%) |  |
| CRP, n (%) | |  | | |
|  | ≦1.0 | 11 (73.3%) | 167 (78.4%) | 0.654 |
|  | >1.0 | 4 (26.7%) | 46 (21.6%) |  |
| GPS, n (%) | |  | | |
|  | 0, 1 | 11 (73.3%) | 182 (84.5%) | 0.242 |
|  | 2 | 4 (26.7%) | 31 (14.5%) |  |
| CONUT score, n (%) | |  | | |
|  | 0-4 | 10 (66.7%) | 161 (75.6%) | 0.454 |
|  | 5-12 | 5 (33.3%) | 52 (24.4%) |  |
| NLR | |  | | |
|  | Median (range) | 1.935 (0.078−4.176) | 2.148 (0.681−23.26) | 0.097 |
| PLR | |  | | |
|  | Median (range) | 135.0 (5.703−371.5) | 160.1 (46.67−1485) | 0.102 |
| AGR | |  | | |
|  | Median (range) | 1.097 (0.638−1.727) | 1.091 (0.511−2.421) | 0.862 |
| Abbreviations are PTPE, percutaneous transhepatic portal vein embolization; ICGR15, indocyanine green retention at 15 min; PD, pancreatoduodenectomy; GPS, Glasgow prognostic score; CONUT, controlling nutritional status; NLR, neutrophil-lymphocyte ratio; PLR, platelet-lymphocyte ratio; AGR, albumin-globulin ratio. | | | | |
